# Improved outcomes for patients undergoing colectomy for acute severe inflammatory colitis by adopting a multi-disciplinary care bundle

**DOI:** 10.1007/s11605-021-05082-2

**Published:** 2021-07-19

**Authors:** D. Boldovjakova, D. S. G. Scrimgeour, C. N. Parnaby, G. Ramsay

**Affiliations:** 1 University of Aberdeen Medical School, Foresterhill, Aberdeen, Scotland; 2 Department of General Surgery, Aberdeen Royal Infirmary, Foresterhill, Aberdeen, Scotland; 3 Health Services Research Unit, University of Aberdeen, Foresterhill, Aberdeen, Scotland

**Keywords:** Inflammatory bowel disease, Crohn’s disease, Ulcerative colitis, Biologics, Colectomy, Multi-disciplinary team, Subtotal colectomy

## Abstract

**Purpose:**

Severe inflammatory colitis as a consequence of inflammatory bowel disease (IBD) may not be amenable to medical management, and surgery is often required. The optimal timing of surgery and perioperative therapeutic care requires a formal link between surgical department and gastroenterology which is often lacking. In this study, we assess the impact of adopting a multidisciplinary care bundle on complication rates of subtotal colectomy in IBD patients.

**Methods:**

This is a single-centre retrospective observational study. Patients were identified through clinical discharge ICD10 codes. Clinical notes of patients who underwent subtotal colectomies from 1 January 2006 to 31 December 2019 were analysed. Socio-demographics, diagnosis, and medical and surgical management data were collected. A multimodule bundle, including weekly MDT discussions, was started in our unit on 1 April 2014. Multivariable logistic regression analysis was performed on these data.

**Results:**

A total of 296 patients were identified with 113 patients of these (38.2%) experiencing a complication post operation. The overall complication rate improved over time (*p* = 0.023). Those patients treated after the initiation of the MDT bundle had reduced complication rates (44.6% versus 33.7%, *p* = 0.045). On multivariate analysis, increasing age (1.023 OR; 95% CI 1.004, 1.041) and procedure performed before MDT bundle (3.1 OR; 95% CI 1.689, 5.723) were independent predictors for post-operative complications.

**Conclusions:**

Closer links between gastroenterology and colorectal specialties have improved patient outcomes in our unit. Whilst IBD MDTs have previously been shown to improve outcomes for patients managed medically, we demonstrate that this interaction, implemented as a multidisciplinary care bundle, also improves surgical outcomes.

## Introduction

Up to one-third of acute colitis patients from Inflammatory Bowel Disease (IBD) will require colectomy having had an inadequate response to medical therapy.^1^ Timely operative intervention is important, as delay increases post-operative complications, including mortality.^2^, ^3^ IBD management has become more complex with the widespread use of rescue immunobiologics.^4^ As a result, it is not possible for IBD care to be managed by a single clinical specialty. However, there is a lack of evidence on whether a multi-disciplinary (MDT) approach for these patients can improve outcomes. We assess the impact of adopting an MDT bundle on early post-operative complication rates of subtotal colectomy in patients with acute colitis. The bundle consisted of a treatment algorithm for patients presenting with acute colitis, weekly IBD MDT meetings and the availability of colorectal surgeons with an interest in IBD.

## Methods

This was a single-centre retrospective observational study. Adult (>16 years) patients were identified through clinical discharge codes. Notes of patients who underwent subtotal colectomies from 01/01/2006 to 31/12/2019 were analysed. Socio-demographics, diagnosis, complications (Clavien-Dindo classification I to V), medical and surgical management data were collected. The MDT care bundle was started on 1^st^ April 2014. Data obtained were analysed using SPSS v26 (IBM, New York). A multivariable logistic regression analysis was modelled using all the parameters in the univariate assessment. The study was registered as an audit of clinical practice with the clinical effectiveness department of NHS Grampian.

## Results

A total of 296 patients were included. Of these, 199 patients were in the pre-bundle group and 97 in the post-bundle group. There were no differences in sex (p=0.109), age (p=0.246), BMI (p=0.569), laparoscopic procedures (p=0.286) and rate of previous operations (p=0.632) between the groups. The number of patients who had biologic medications before their operation was significantly higher in the post-bundle group (n=38; 39%) when compared to the pre-bundle group (n=21; 11%; p<0.0001). Patients treated in the post-bundle group had significantly reduced overall complication rates (44.6% versus 33.7%, p=0.045. The rates of severe complications (Clavien Dindo III/IV/V) had also reduced significantly between the pre-bundle (n=36; 18%) and the post-bundle (n=9; 9.3%) (p=0.021) (Table 1). On multivariate analysis, increasing age [1.023 OR; 95% CI 1.004, 1.041] and procedure performed before MDT bundle [3.1 OR; 95% CI 1.689, 5.723] were independent predictors for post-operative complications (Table 2).

**Table 1:** Complication profile before and after the start of the IBD care bundle, by Clavien Dindo

	Before Bundle (n=199)	%	After bundle (n=97)	%	p	Total
Patients with no complications	112	56.3	63	65.0	0.045	175
Patients with complication	87	43.7	34	35.0		121
Clavien Dindo						
1	1	1.1	2	5.9	0.255	3
2	50	57.5	23	67.6		73
3a	8	9.2	3	8.8		11
3b	21	24.1	5	14.7		26
4	3	3.4	0	0.0		3
5	4	4.6	1	2.9		5

**Table 2:** Multivariable model for complication analysis results

			95% CI		
		Odds Ratio	Low	High	p value
Sex	Male	Ref			
	Female	0.85	0.48	1.5	0.577
Age	(by year)	1.023	1.004	1.041	*0.014*
Biologic use	No biologic	Ref			
	Biologic	1.417	0.693	2.9	0.339
MDT start	After	Ref			
	Before	3.1	1.689	5.723	*<0.001*
Colitis type	Crohn’s	Ref			
	Ulcerative colitis	2.233	0.827	6.031	0.113
	Indeterminate	1.207	0.693	2.898	0.634
Urgency	Emergency	Ref			
	Urgent	1.727	0.938	3.177	0.089

## Discussion

This work demonstrates that the rates of complications after IBD colectomies in the North of Scotland have improved since 2014 following the introduction of an MDT care bundle. This was more than merely a weekly MDT discussion and allowed for a consistent timepoint of surgical referral to surgeons with an interest in IBD. Whilst we cannot apportion causality, this improvement is contemporaneous with the establishment of a formalised IBD MDT working process and the adoption of a care bundle in our tertiary centre. Despite the increased use of immunobiologics, not only has the overall rate of complications reduced, but the rate of severe complications has also decreased.

The literature on IBD MDT has previously concentrated on non-operative outcomes such as timing of diagnosis, flare-up detection rates, side effects of medical therapies and time to surgery as primary end points.^5^, ^6^ However, this study has found that an MDT care bundle can improve surgical outcomes in patients with IBD. We propose that, through optimal referral to the surgical team and early awareness of individuals who are starting rescue therapy to the IBD colorectal surgeon, patients who proceed to an operation do so in a more controlled and organised way and this leads to improved surgical outcomes.
